# Phytotherapeutic potential against MRSA: mechanisms, synergy, and therapeutic prospects

**DOI:** 10.1186/s13020-024-00960-8

**Published:** 2024-06-22

**Authors:** Qiqi He, Julie Meneely, Irene R. Grant, Jason Chin, Séamus Fanning, Chen Situ

**Affiliations:** 1 Institute for Global Food Security, School of Biological Sciences, Queen’s University Belfast, Belfast, BT9 5DL UK; 2 University College Dublin Centre for Food Safety, School of Public Health, Physiotherapy & Sports Science, University College Dublin, Dublin, Republic of Ireland

**Keywords:** MRSA, Antimicrobial resistance, Phytotherapeutics, Medicinal plants, *Rheum palmatum* L., Pseudo-multicellular cells, Morphological changes

## Abstract

**Background:**

Rising resistance to antimicrobials, particularly in the case of methicillin-resistant *Staphylococcus aureus* (MRSA), represents a formidable global health challenge. Consequently, it is imperative to develop new antimicrobial solutions. This study evaluated 68 Chinese medicinal plants renowned for their historical applications in treating infectious diseases.

**Methods:**

The antimicrobial efficacy of medicinal plants were evaluated by determining their minimum inhibitory concentration (MIC) against MRSA. Safety profiles were assessed on human colorectal adenocarcinoma (Caco-2) and hepatocellular carcinoma (HepG2) cells. Mechanistic insights were obtained through fluorescence and transmission electron microscopy (FM and TEM). Synergistic effects with vancomycin were investigated using the Fractional Inhibitory Concentration Index (FICI).

**Results:**

*Rheum palmatum* L., *Arctium lappa* L. and *Paeonia suffructicosaas* Andr. have emerged as potential candidates with potent anti-MRSA properties, with an impressive low MIC of 7.8 µg/mL, comparable to the 2 µg/mL MIC of vancomycin served as the antibiotic control. Crucially, these candidates demonstrated significant safety profiles when evaluated on Caco-2 and HepG2 cells. Even at 16 times the MIC, the cell viability ranged from 83.3% to 95.7%, highlighting their potential safety. FM and TEM revealed a diverse array of actions against MRSA, such as disrupting the cell wall and membrane, interference with nucleoids, and inducing morphological alterations resembling pseudo-multicellular structures in MRSA. Additionally, the synergy between vancomycin and these three plant extracts was evident against MRSA (FICI < 0.5). Notably, aqueous extract of *R. palmatum* at 1/4 MIC significantly reduced the vancomycin MIC from 2 µg/mL to 0.03 µg/mL, making a remarkable 67-fold decrease.

**Conclusions:**

This study unveil new insights into the mechanistic actions and pleiotropic antibacterial effectiveness of these medicinal plants against resistant bacteria, providing robust evidence for their potential use as standalone or in conjunction with antibiotics, to effectively combat antimicrobial resistance, particularly against MRSA.

## Background

With a global median resistance level of 35%, Methicillin-resistant *Staphylococcus aureus* (MRSA) is acknowledged as one of the priority pathogens worldwide [1]. The World Health Organization has declared an urgent need for the development of novel antimicrobial compounds and antibiotic alternatives to safeguard the efficacy of existing antibiotics in a race to combat the emergence and spread of antimicrobial resistance (AMR), one of the world’s most pressing health and environmental issues. This has sparked a renewed interest in exploring medicinal plants as potential antimicrobial agents, or as supplementary phytotherapy in the management of infectious disease [2, 3]. The use of whole plants or plant mixtures to cure diverse ailments has a long history that predates the Palaeolithic era, and this practice persists in many parts of the world today. Undoubtedly, mounting evidence from both in vitro and in vivo studies has demonstrated the pharmacological and therapeutic superiority of crude plant preparations over single constituents [4]. The multifactorial effects of plant-based therapies are believed to arise from the presence of a vast array of phytochemicals within the plant, which can target multiple bacterial sites simultaneously [5]. Cutting-edge research has uncovered the remarkable ability of phytochemicals to sensitize and potentiate the effectiveness of antibiotics, as well as possessing high susceptibility to resistant bacteria with low toxicity towards host cells [6].

Plants possessing medicinal properties represent an important component of the pharmaceutical industry due to their attributes and effectiveness in disease prevention and treatment. The medicinal plants selected for this study have a long history of traditional use in management of a diverse array of infectious diseases [7]. In the present study, we evaluate the antibacterial activity of aqueous preparations of these plants against MRSA (NCTC 12493), individually and/or in combination with vancomycin, the last resort of antibiotic for this deadly resistant pathogen. The safety profile of the candidate plants was assessed for their potential cytotoxicity on mammalian cells. Consequently, we employed both fluorescence microscopy (FM) and transmission electron microscopy (TEM) to determine the mechanistic principles underlying the efficacy of these medicinal plants, with respect to morphological and ultrastructural changes in MRSA. This is the first report that illustrates the subcellular structural alterations that occur in MRSA following exposure to medicinal plant extracts.

## Methods

### Bacterial strains and growth conditions

The MRSA (NCTC 12493) used in this study was supplied by the National Collection of Type Cultures (Public Health England, Colindale, London, UK). Mueller–Hinton (MH) agar or Mueller–Hinton (MH) broth was employed for preculturing and preparing the bacterial suspension for MRSA.

### Preparation of medicinal plant extracts

The 68 dried commercial medicinal plants with specific parts of plant used for this study are listed in Table 1. The processing of plant materials involved grinding the dried medicinal plants into fine powder using a planetary ball mill (PM100, Retsch, UK). The fine plant material was suspended in distilled water at 1:1000 (w/v) and subjected to ultrasonic treatment (VWR Ltd, UK) at 45 kHz for 15 min, followed by immersion in a boiling water bath for an additional 30 min. The resulting extract was centrifuged at 12,000 rpm for 10 min at room temperature (RT) and the supernatant was filtered through a 0.45 μm syringe filter (Merck Millipore Ltd, Ireland) before testing.

**Table 1:** Medicinal plants (n = 68) screened for antibacterial activity against MRSA (NCTC 12493) and their MIC (mg/mL)

No	Scientific name	Common name	Chinese name	Part of plant	MIC (mg/mL) of MRSA (NCTC 12493)
1	*Rheum palmatum* L.	*Chinese rhubarb*	*Dahuang*	Root	0.0078
2	*Arctium lappa* L.	*Great burdock achene*	*Niubangzi*	Fruit	0.0078
3	*Paeonia suffructicosa* Andr.	*Tree peony*	*Mudanpi*	Bark	0.0078
4	*Reynoutria japonica* Houtt.	*Asian knotweed*	*Huzhang*	Root	0.0156
5	*Cyrtomium fortunei* J.Smith	*Fortunei's holly-fern*	*Guanzhong*	Root	0.0156
6	Sargentodoxa cuneata (Oliv.) Rehder & E.H.Wilson	*Sargentgloryvine*	*Hongteng*	Root and stem	0.0156
7	*Agrimonia pilosa* Ledeb.	*Hairy agrimony*	*Xianhecao*	Leaf	0.0312
8	*Smilax china* L.	*China root*	*Baqia*	Root	0.0312
9	*Lithospermum erythrorhizon* Sieb. et Zucc.	*Purple gromwell*	*Zicao*	Root	0.0312
10	*Pulsatilla chinensis (Bunge)* Regel	*Chinese Pulsatilla Root*	*Baitouweng*	Root	0.0312
11	*Sanguisorba officinalis* L.	*Burnet bloodwort*	*Diyu*	Root	0.0390
12	*Chrysanthemum indicum* L.	*Flos Chrysanthemi*	*Yejuhua*	Flower bud	0.0625
13	*Forsythia suspensa (Thunb.)* Vahl	*Weeping forsythia*	*Lianqiao*	Fruit	0.0625
14	Cornus officinalis Siebold & Zucc.	*Corni fructus*	*Shanzhuyu*	Fruit	0.0781
15	*Corydalis bungeana* Turcz.	*Corydalis bungeanae herba*	*Diding*	Whole parts	0.0781
16	*Ophiopogon japonicus* (Thunb.) Ker-Gawl	*Dwarf lilyturf*	*Maidong*	Root	0.0781
17	*Magnolia officinalis* Rehder & E.H.Wilson	*Danghoobak*	*Houpu*	Stem peel	0.0781
18	*Taxillus sutchuenensis (Lecomte) Danser*	*Taxillus*	*Sangjisheng*	Stem	0.0781
19	*Taraxacum mongolicum* Hand.-Mazz.	*Herba Taraxaci*	*Pugongying*	Whole parts	0.1250
20	*Pogostemon cablin* (L.) H.S.Irwin & Barneby	*Herba Pogostemonis*	*Guanghuoxiang*	Leaf	0.1250
21	*Houttuynia cordata* Thunb	*Chameleon*	*Yuxingcao*	Leaf and stem	0.1250
22	*Lobelia Chinensis* Lour	*Mizo-kakushi*	*Banbianlian*	Whole parts	0.1250
23	*Senecio scandens* Buch.-Ham. ex D. Don	*Senecio scandens*	*Qianliguang*	Whole parts	0.2500
24	*Lonicera japonica* Thunb	*Honeysuckle*	*Jinyinhua*	Flower bud	0.2500
25	*Paeonia lactiflora* Pall	*Red peony root*	*Chishao*	Root	0.2500
26	*Stellaria dichotoma var. lanceolata* Bunge	*Radix stellariae*	*Chaihu*	Root	0.3125
27	*Spirodela polyrhiza* (L.) Schleid	*Common duckweed*	*Fuping*	Root	0.3125
28	*Allium macrostemon* Bunge	*Allii macrostemonis bulbus*	*Xiebai*	Stem	0.3125
29	*Actaea cimicifuga* L.	*Black cohosh rhizome*	*Shengma*	Stem and root	0.3125
30	*Juncus effusus* L.	*Common rush*	*Dengxincao*	Stem	0.3125
31	*Platycodon grandiflorus* (Jacq.) A.DC.	*Ballonblume*	*Jiegeng*	Root	0.6250
32	*Isatis tinctoria* subsp. *tinctoria*	*Indigowoad Leaf*	*Daqingye*	Leaf	1.0000
33	*Scutellaria barbata* D. Don	*Barbed skullcap*	*Banzhilian*	Stem and leaf	1.0000
34	*Coptis chinensis* Franch.	*Coptis chinensis*	*Huanglian*	Root	1.0000
35	*Neolitsea cassia* (L.) Kosterm	*Cinnamon*	*Rougui*	Stem peel	1.0000
36	*Scutellaria baicalensis* Georgi	*Baical skullcap*	*Huangqin*	Root	> 1.000
37	*Artemisia argyi* H.Lév. & Vaniot	*Artemisiae argyi folium*	*Aiye*	Leaf	> 1.000
38	*Lsatis tinctoria* L.	*Dyer’s Woad*	*Banlangen*	Root and leaf	> 1.000
39	*Patrinia scabiosifolia* Link	*Herba patriniae*	*Baijiangcao*	Whole parts	> 1.000
40	*Scleromitrion diffusum (Willd.)* R.J.Wang	*Hedyotis diffusa*	*Baihuasheshecao*	Whole parts	> 1.000
41	*Pteris multifida* Poir	*Pteris multifidae herba*	*Fengweicao*	Whole parts	> 1.000
42	*Iris domestica* (L.) Goldblatt and Mabb	*Blackberry lily*	*Shegan*	Root	> 1.000
43	*Dolomiaea costus* (Falc.) Kasana & A.K.Pandey	*Costus*	*Muxiang*	Root	> 1.000
44	*Portulaca oleracea* L.	*Common purslane*	*Machimi*	Whole parts	> 1.000
45	*Coix lacryma-jobi var. ma-yuen* (Rom.Caill.) Stapf	*Coicis semen*	*Yiren*	Fruit	> 1.000
46	*Fraxinus chinensis* Roxb	*Ash bark*	*Qinpi*	Stem peel	> 1.000
47	*Sophora tonkinensis* Gagnep	*Sophora tonkinensisi*	*Shandougen*	Root and stem	> 1.000
48	*Areca catechu* L.	*Areca peel*	*Dafupi*	Fruit peel	> 1.000
49	*Reynoutria multiflora* (Thunb.) Moldenke	*Caulis polygoni multiflori*	*Heshouwu*	Root	> 1.000
50	*Artemisia caruifolia* var. caruifolia	*Artemisiae apiaceae berba*	*Qinghao*	Stem and leaf	> 1.000
51	*Setaria italica* (L.) P.Beauv	*Setaria viridis*	*Gouweicao*	Whole parts	> 1.000
52	*Sphaerophysa salsula* (Pall.) DC.	*Austrian peaweed*	*Niaopaocao*	Fruit	> 1.000
53	*Prunella vulgaris* L.	*Brunelle vulgaire*	*Xiakucao*	Fruit	> 1.000
54	*Atractylodes lancea* (Thunb.) DC.	*Atractylodes*	*Cangshu*	Stem and root	> 1.000
56	*Lysimachia christinae* Hance	*Christina loosestrife*	*Qinqiancao*	Whole parts	> 1.000
57	*Gentiana scabra* Bunge	*Chinese gentian*	*Longdancao*	Stem and root	> 1.000
58	*Epimedium sagittatum (Siebold & Zucc.) Maxim.*	*Epimedii folium*	*Yingyanghuo*	Leaf	> 1.000
59	*Platycladus orientalis* (L.) Franco	*Cacumen platycladi*	*Cebaiye*	Leaf	> 1.000
60	*Gentiana macrophylla* Pall.	*Large-leaf gentian*	*Qinjiao*	Root	> 1.000
61	*Panax notoginseng* (Burkill) F.H.Chen	*Notoginseng radix*	*Tianqi*	Stem and root	> 1.000
62	*Pueraria montana var. lobata* (Willd.) Maesen & S.M.Almeida ex Sanjappa & Predeep	*Puerariae lobatae radix*	*Gegen*	Root	> 1.000
63	*Paeonia lactiflora* Pall.	*Radix paeoniae alba*	*Baishao*	Root	> 1.000
64	*Sedum sarmentosum* Bunge	*Stringy stonecrop*	*Chuipencao*	Whole parts	> 1.000
65	*Anemarrhena asphodeloides* Bunge	*Anemarrhena rhizome*	*Zhimu*	Stem and root	> 1.000
66	*Asparagus officinalis* L.	*Asparagus*	*Baibu*	Root	> 1.000
67	*Xanthium strumarium* subsp. *strumarium*	*Rough cocklebur*	*Cangerzi*	Fruit	> 1.000
68	*Trichosanthes kirilowii* Maxim.	*Trichosanthis radix*	*Tianhuafen*	Root	> 1.000

### Anti-MRSA activity screening

Following the guidelines set by the British Society for Antimicrobial Chemotherapy (BSAC), the microdilution method was employed to determine both MBC and MIC [8]. Extracts of plants and antibiotic vancomycin (CAS 1404-93-9; Sigma-Aldrich Ltd, Poole, UK) were serially diluted in MH broth at concentrations ranging from 0.9 to 1000 mg/L and 0.25 to 128 mg/L, respectively, and added to microtiter plates (M2311-100EA, Greiner, UK). The extracts exhibited a subtle green or yellow tint, which did not obstruct or interfere with the observation of bacterial growth manifested as turbidity within the well.

MRSA suspensions, initially adjusted to McFarland standards (0.5), were diluted at a ratio of 1:100 in MH broth. A sterile (negative) control containing MH broth only, a growth control with a bacterial suspension, and a positive control with vancomycin were included in each 96-well plate. The plates were incubated for 18–20 h at 37 °C. The MIC value was established as the lowest concentration of the test sample that completely inhibited bacterial growth, confirmed by the absence of visible growth under the specified experimental conditions. The determination of MBC involved sub-culturing 10 μL of each dilution from and above MIC wells and spotting onto Mueller–Hinton (MH) agar (Oxoid, CM0337; Hampshire, UK) plate and incubated for a further 24 h at 37 °C. The MBC was defined as the lowest micro-dilution of antimicrobial compound that prevents organism growth on the agar plate, with a 99.9% killing (3 log reduction) in CFU/mL compared to untreated organism (growth control) [9].

The kinetics of bacterial growth were studied to evaluate the efficacy of medicinal plant extracts in inhibiting bacteria. This assessment was carried out at three concentrations (MIC, 2MIC, and 4MIC) at 37 °C, using a microplate reader (SynergyTM HT, BioTek, Winooski, Vermont, USA) for accurate measurements. The OD at 600 nm of each well was automatically measured and recorded every 30 min over 24 h. Data were acquired using Gen5 1.10 software, exported to Microsoft Excel for processing, and expressed as the mean value of three replicates. Analysis was conducted using Microsoft Excel and GraphPad Prism 7.0 (GraphPad Software Inc., San Diego, CA, USA). Normalization for comparison involved aligning the same starting point of all datasets, with different values shown on the y-axis for each sample.

The time-kill assay was carried out to determine the rate and extent of microbial killing over time by the plant-derived substances. A bacterial suspension (1 × 10^6^ CFU/mL) was treated with a medicinal plant extract (500 μL) at concentrations of 1, 2, 3 and 4 × MIC (7.8 μg/mL, 15.6 μg/mL, 23.4 μg/mL, 31.2 μg/mL) of *R. palmatum, Arctium lappa* L. and *P. suffructicosa*, respectively. The mixture was incubated at 37 °C with gentle agitation in a Labwit shaker (ZWY-100H, Australia). Samples (10 μL) were taken at 0, 1, 3, 6, 20 and 24 h, serially diluted, and plated on MH agar. After incubation for 24 h at 37 °C, a bactericidal effect was defined as a 3-log reduction in viable cell count. The time-kill assays were performed in triplicate, and GraphPad Prism 7.0 was used for graphical representation.

### Evaluation of the synergistic effect

A broth checkerboard microdilution assay was employed to investigate combined treatments of vancomycin and plant extracts [10]. The assay featured a two-dimensional checkerboard with two-fold dilutions of the antibiotic vancomycin (0.25–128 mg/L) horizontally and plant extract (0.9–1000 mg/L) vertically. The bacterial suspension (1 × 10^6^ CFU/mL) was added and incubated for 18–20 h at 37 °C in 96-well plates. Controls included bacterial suspension, antibiotics and plant dilution controls. The MIC was determined to be the lowest concentration of plant extracts and antibiotic without visible bacterial growth. Effective combinations were identified, and the fractional inhibitory concentration (FIC) was calculated for the first clear well containing both antimicrobial agents:

FIC of A (plant extract) = MIC_A+B_ in combination/ MIC_A_ alone.

FIC of B (antibiotic) = MIC_B+A_ in combination/ MIC_B_ alone.

The FIC index (FICI), the sum of both calculated FIC values, was interpreted as follows: synergistic (≤ 0.5), additive (> 0.5 and ≤ 1); indifferent (> 1 and ≤ 4); antagonistic (> 4) [10].

### Cytotoxicity evaluation of potential plant candidates

The cytotoxicity of plant extracts against hepatocellular carcinoma (HepG2) and colorectal adenocarcinoma (Caco-2) cell lines was assessed using the MTT (3-(4, 5-dimethylthiazolyl-2)-2, 5-diphenyltetrazolium bromide) colorimetric assay [11]. Cell lines were subcultured at 37 °C in a CO_2_ incubator with 5% CO_2_ gas and 95% humidity, using minimal essential medium (MEM) supplemented with 10% fetal bovine serum, 1% penicillin–streptomycin, 1 mM sodium pyruvate, and 2 mM L-glutamine. All reagents used were purchased from Life Technologies (Paisley, Scotland, UK). Cell suspension (100 μL) was seeded into BD Falcon 96-well microtiter plates (BD Biosciences, US) at a density of 1 × 10^4^ cells/well and 5 × 10^3^ cells/well for HepG2 and Caco-2 cells, respectively. After a 24 h cell attachment period, plant extracts (100 μL) at dilutions ranging from 0.0002 to 10 mg/mL were applied and incubated for 48 h. Following cell washing with phosphate buffered saline (PBS), 50 μL of 2 mg/mL MTT solution was added. After a 4 h incubation, the supernatant was discarded, and 200 μL of preheated dimethyl sulfoxide (DMSO) was introduced to dissolve formazan crystals. The plate was incubated at 37 °C with agitation for a further 10 min. Absorbance was measured at 570 nm with a reference filter at 630 nm using a TECAN microtiter plate reader (Safire II, BASIC). Viability was determined by calculating the percentage of sample absorbance relative to the untreated control. According to ISO 10993-5, the standard for test in vitro cytotoxicity, cell viability percentages less than 40% indicate significant cytotoxicity, values between 40 and 60% suggest moderate cytotoxic effects. Viability within the range of 60% to 80% is considered weak, and percentages above 80% are classified as non-cytotoxic [11].

### FM analysis

Cultures of MRSA in the exponential phase were used to prepare a cell suspension at a concentration of 1 × 10^6^ CFU/mL. Subsequently, 500 μL of each medicinal plant extracts of *R. palmatum*, *A. lappa* and *P. suffructicosa* at their MIC (7.8 μg/mL) diluted in MH broth was introduced into the prepared bacterial cell suspension (500 μL). The mixture was then incubated at 37 °C for 20 h before applying fluorescent dyes. As a growth control, a 500 μL cell suspension in 500 μL of MH broth was used. Following incubation, cells were rinsed, pelleted, and treated with 20 μl solution of 4% (v/v) formaldehyde for 10 min at room temperature in the absence of light. For nucleus staining, 20 μl Hoechst 33342 (Life Technologies, Scotland, UK) at 2 μM was added and incubated with the cells for 10 min at room temperature (RT) followed by three washes with PBS. Subsequently, the cells were counterstained with the red membrane dye FM 4–64 64 (Thermo Fisher Scientific, UK) at a concentration of 5 μg/mL for 5 min at RT, followed by additional 2 min on ice. The staining process was protected from light. FM images were captured immediately using the *Olympus BX63* fluorescent microscope equipped with an *Olympus DP74* camera with an excitation wavelength of 515 nm and an emission wavelength of 640 nm for the membrane, Excitation/Emission: 361/497 for the nuclear images, and co-staining using *CellSens Dimension* imaging acquisition software (Olympus, Center Valley, USA).

### TEM analysis

Following a 24 h exposure to the medicinal extracts of *R. palmatum*, *A. lappa* and *P. suffructicosa*, bacterial cells were fixed for 2 h at RT in 2.5% (v/v) glutaraldehyde and 1.5% (v/v) paraformaldehyde buffered in PHEM (pH 7). The PHEM buffer comprised of 60 mM PIPES (piperazine-*N, N'*-bis), 25 mM HEPES (4-(2-hydroxyethyl)-1-piperazineethanesulfonic acid), 10 mM EGTA (ethylene glycol-bis(β-aminoethyl ether)-*N,N,N',N'*-tetraacetic acid) and 2 mM MgCl_2_. Afterward, the cells underwent three times washes with PHEM buffer and resuspended in a 2% agarose solution. The agarose embedding technique, typically employed for tissue sample preparation, was repurposed with slight modifications for the current study to preserve bacterial cell integrity and ensure optimal image quality. The embedded cell pellets were processed using an automated tissue processor Leica EM-TP. Samples were first rinsed in PHEM buffer, then fixed, and stained with 1% (v/v) osmium tetroxide for 45 min, followed by dehydrated with a sequential ethanol series (30%, 50%, 75%, 95%, 3 × 100%), and finally three times washes in 100% acetone before embedding in Spurr resin (Electron Microscopy Sciences, Hatfield, USA). Semi-thin Sects (1 μm) were cut with a glass knife, followed by further cutting into ultrathin Sects. (90 nm) using a diamond knife via Ultracut-UCT ultramicrotome (Leica Microsystems, Vienna, Austria). Ultrathin sections were placed on formvar-coated, 300-mesh copper grids, and post-stained in the 2% (w/v) uranyl acetate and 1% (v/v) osmium tetroxide. The micrographs were captured using TEM (Jeol JEM-1400, USA), operated at 80 kV and magnification between × 1000 and × 50,000.

## Results

### Anti-MRSA activity of aqueous plant extracts

The MICs of 68 crude aqueous plant preparations against MRSA (NCTC 12493) are shown in Table 1. Eight plants exhibited significant anti-MRSA efficacy, displaying MIC values ranging from 7.8 μg/mL to 31.2 μg/mL. The minimum bactericidal concentrations (MBCs) for all eight plants extract a four-fold increase compared to their respective MICs, except for *Reynoutria japonica Houtt* (8 × MIC) (Table 2). The positive control, vancomycin, displayed MIC and MBC values of 2 µg/mL and 8 µg/mL (4 × MIC), respectively.

**Table 2:** The MBC values of eight medicinal plant extracts with lowest MICs against MRSA

Medicinal plants	Antibacterial activity against MRSA (NCTC 12493)	
	MIC (mg/mL)	MBC (mg/mL)
Vancomycin	0.0002	0.0008
*R. palmatum*	0.0078	0.0312
*A. lappa*	0.0078	0.0312
*P. suffructicosa*	0.0078	0.0312
*R. japonica*	0.0156	0.1248
*C. fortunei*	0.0156	0.0624
*A. pilosa*	0.0312	0.1248
*S. china*	0.0312	0.1248
*L.erythrorhizon*	0.0312	0.1248

**Fig. 1:**
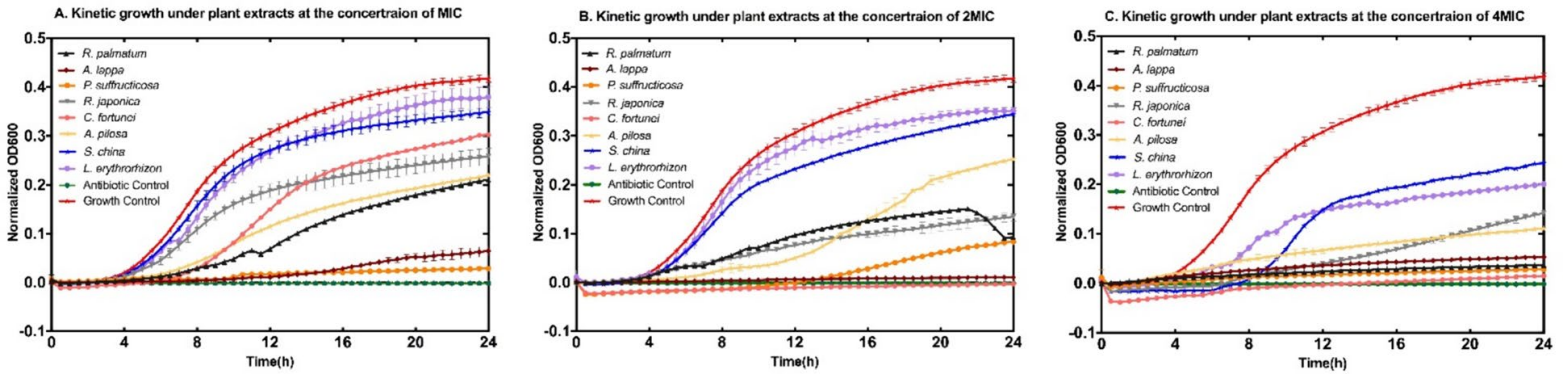
Kinetic growth curves of MRSA (NCTC 12493) treated with eight aqueous medicinal plant extracts against MRSA (NCTC 12493). **A** MIC, **B** 2MIC, and **C** 4MIC. Antibiotic control: vancomycin at MIC. Growth control: MRSA suspension in MH broth

The growth inhibition effectiveness of these eight medicinal plant extracts were monitored over a 24 h period (Fig. 1). Treatment with *Paeonia suffructicosa* Andr. and *Arctium lappa* L. at their respective MICs resulted in pronounced growth inhibition, characterized by relatively flat curves compared to vancomycin, the antibiotic control (Fig. 1B). Whereas at 4 × MICs (i.e. MBCs), *R.* *palmatum,* *P.* *suffructicosa*, *A.lappa* and *Cyrtomium fortunei* J. Smith exhibited the strongest anti-MRSA effect, similar to the antibiotic control of vancomycin.

**Fig. 2:**
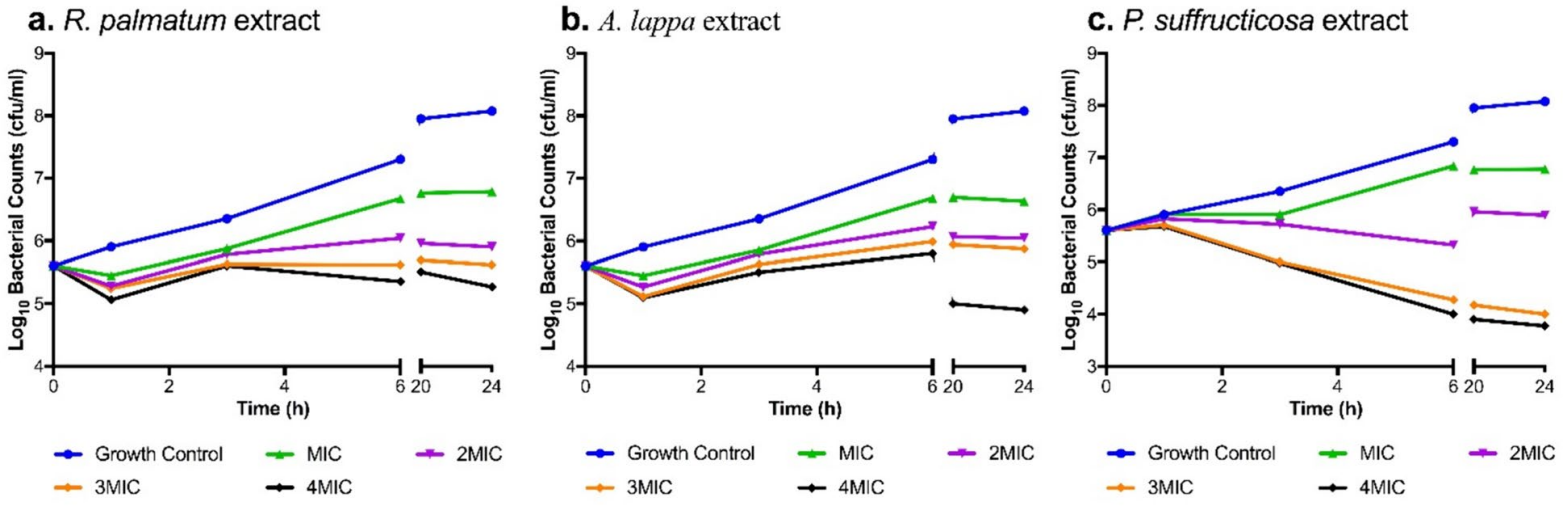
Log10 total viable bacterial colonies after treatment with *R. palmatum *(**a**)*, A. lappa * (**b**) and *P. suffructicosa * (**c**) extract at 1, 2, 3 and 4 × MIC for 0, 1, 3, 6, 20 and 24 h against MRSA (NCTC 12493)

Time-kill assay revealed the bactericidal activity of three plants possessed against MRSA (NCTC 12493): *R. palmatum, Arctium lappa* L. and *P. suffructicosa*. The bactericidal properties of the medicinal plant extracts at 1, 2, 3 and 4 times the MIC are outlined in Table 3, presenting the log reduction in viable bacterial cell count following a 24 h treatment period. As shown in Fig. 2, treatment with *R. palmatum* (Fig. 2a) or *A. lappa* (Fig. 2b) at doses ≥ 2 × MICs resulted in similar growth patterns, manifesting an initial decline of 1-log within the first hour and a 3-log reduction (at 4 × MICs) after 24 h compared to the growth control. Whereas exposure to *P. suffructicosa* (Fig. 2c) at ≥ 3 × MIC induced a significant reduction of viable MRSA after 1 h incubation followed with a fast downward trend of the growth curve, achieving > 3-log reduction by 6 h.

**Table 3:** Log reduction of MRSA in the presence of *R. palmatum*, *A. lappa* and *P. suffructicosa*

Time (Hours)	Log reduction^a^ with the treatment of* R. palmatum*				Log reduction^a^ with the treatment of* A. lappa*				Log reduction^a^ with the treatment of *P. suffructicosa*			
	MIC	2MIC	3MIC	4MIC	MIC	2MIC	3MIC	4MIC	MIC	2MIC	3MIC	4MIC
0	< 1	< 1	< 1	< 1	< 1	< 1	< 1	< 1	< 1	< 1	< 1	< 1
1	< 1	1	1	1	< 1	1	1	1	< 1	< 1	< 1	< 1
3	< 1	1	1	1	1	1	1	1	< 1	1	1	1
6	1	1	2	2	1	1	1	2	1	2	3	3
20	1	2	2	2	1	2	2	3	1	2	3	> 4
24	1	2	2	3	1	2	2	3	1	2	> 4	> 4

^a^1-log reduction = 90% kill; 2-log reduction = 99% kill; 3-log reduction = 99.9% kill

Log reduction of MRSA (NCTC 12493) in the presence of 1, 2, 3, and 4 × minimum inhibitory concentration (MIC) of *R. palmatum*, *A. lappa* and *P. suffructicosa* infusions at 0, 1, 3, 6, 20 and 24 h

### Synergistic interactions between medicinal plants and the antibiotic vancomycin

Eight medicinal plants exhibiting promising anti-MRSA activity were chosen for a combination study with antibiotic vancomycin, and a summary of the results is provided in Table 4. A synergistic effect was noted between vancomycin and three plant extracts, *R. palmatum*, *Lithospermum erythrorhizon* Sieb. et Zucc. and *A. pilosa*, with FIC indices of 0.25, 0.28 and 0.5, respectively.

**Table 4:** Effects of the combination between vancomycin and medicinal plant extracts against MRSA (NCTC 12493)

Medicinal plants	MIC alone (mg/mL)		MIC in combination (mg/mL)		FICI	Effect
	Plant	Vancomycin	Plant	Vancomycin		
*R. palmatum*	0.0078	0.002	0.0019	0.00003	0.25	Synergy
*A. lappa*	0.0078	0.002	0.0019	0.0020	1.24	Indifference
*P. suffructicosa*	0.0078	0.002	0.1250	0.0010	16.52	Antagonism
*R. japonica*	0.0156	0.002	0.0039	0.0020	1.25	Indifference
*C. fortunei*	0.0156	0.002	0.2500	0.00006	16.05	Antagonism
*A. pilosa*	0.0312	0.002	0.0078	0.0005	0.50	Synergy
*S. china*	0.0312	0.002	0.0078	0.0020	1.25	Indifference
*L.erythrorhizon*	0.0312	0.002	0.0078	0.00006	0.28	Synergy

### Safety profile of antibacterial plant candidates towards mammalian cells

**Fig. 3:**
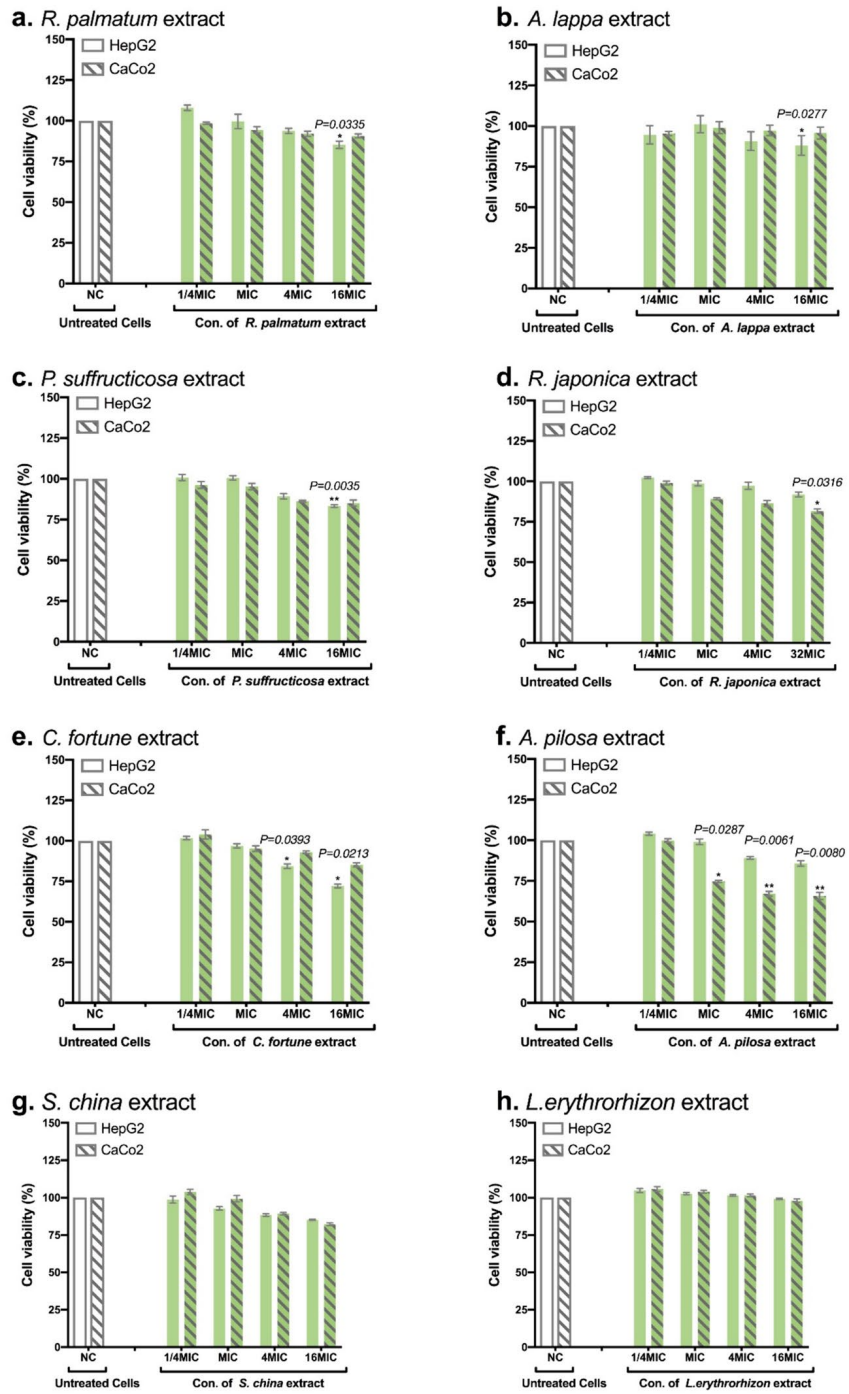
Viability of HepG2 and Caco-2 cells was assessed using MTT after treatment with medicinal plants. Plant extractions included **a** *R. palmatum*, **b** *A. lappa*, **c** *P. suffructicosa*, **d** *R. japonica*, **e** *C. fortune*, **f** *A. pilosa*, **g** *S. china*, **h** *L.erythrorhizon*. Four times the MBC equals 16 × MIC for all plant extracts except *R. japonica,* where it is 32 × MIC. Error bars represent one standard deviation (SD) from the mean. Statistical analyses were conducted using ordinary one-way ANOVA, Dunnett’s multiple comparisons test in Graph Pad Prism 7 software. Statistical significance was defined as p values below 0.05, denoted as * for p < 0.0332 (*) and ** for p < 0.0021 (**)

Table 5 illustrates the cell viability resulting from the application of plant extracts at four different concentrations (i.e. 1/4 × MIC, MIC, 4 × MIC, 16 × MIC). HepG2 cells remained remarkable viability even at elevated concentrations up to 16 × MIC (equivalent to 4 × MBC) from different plants, ranging from 88.3% to 101.5%. Exceptions were observed for *C. fortune* (Fig. 3e), with viability recorded at 84.3% at 4 × MIC and 72.1% at 16 × MIC, and *P. suffructicosa* with 83.3% at 16 × MIC (Fig. 3c). When testing on Caco-2 cells, *A. pilosa* exhibited 74.7% viability at MIC which was further reduced to 67.1% (4 × MIC) and 65.7% (16 × MIC), respectively (Fig. 3f). Whereas application of *R. japonica* (Fig. 3d) and *S. china* (Fig 3g) at 4 × MBC resulted in a cell viability of 81.5% and 82.2%, respectively. Based on the ISO 10993–5 standard for test in vitro cytotoxicity [12], there was no observed cytotoxicity activity in any of the tested plant extracts at their concentrations ranging from MIC to 8 × MIC, except for *A. pilosa,* which displayed weak cytotoxicity on Caco-2 cells without a distinct dose-dependent pattern.

**Table 5:** Viability (%) of HepG2 and Caco-2 cells treated with medicine plant extracts

Plant	Concentration		Cell Viability (%)	
	MIC/MBC	ug/mL	HepG2	Caco-2
*R. palmatum*	1/4MIC	1.95	94.7	95.4
	MIC	7.8	101.2	99.0
	4MIC	31.2	90.7	97.2
	16MIC	124.8	88.1	95.7
*A. lappa*	1/4MIC	1.95	107.9	98.5
	MIC	7.8	99.6	94.3
	4MIC	31.2	93.8	92.0
	16MIC	124.8	85.3	90.7
*P. suffructicosa*	1/4MIC	1.95	100.8	96.2
	MIC	7.8	100.6	95.3
	4MIC	31.2	89.3	86.2
	16MIC	124.8	83.3	85.1
*R. japonica*	1/4MIC	3.9	102.3	99.0
	MIC	15.6	98.7	89.2
	4MIC	62.4	97.2	86.4
	32MIC	499.2	91.9	81.5
*C. fortunei*	1/4MIC	3.9	101.7	103.9
	MIC	15.6	97.0	95.3
	4MIC	62.4	84.3	92.9
	16MIC	249.6	72.1	85.2
*A. pilosa*	1/4MIC	7.8	104.1	99.8
	MIC	31.2	99.0	74.7
	4MIC	124.8	89.2	67.1
	16MIC	499.2	85.8	65.7
*S. china*	1/4MIC	7.8	98.7	103.8
	MIC	31.2	92.8	99.3
	4MIC	124.8	88.3	89.2
	16MIC	499.2	85.3	82.2
*L. erythrorhizon*	1/4MIC	7.8	104.7	105.8
	MIC	31.2	102.7	104.0
	4MIC	124.8	101.5	101.5
	16MIC	499.2	99.3	97.9

A comparison of the effects of four concentrations of medicinal plant extracts on cell viability (%) using MTT assays over a 48 h period on HepG2 and Caco-2 cell lines for cytotoxicity assessment

### Morphological and ultrastructural alterations of MRSA

**Fig. 4:**
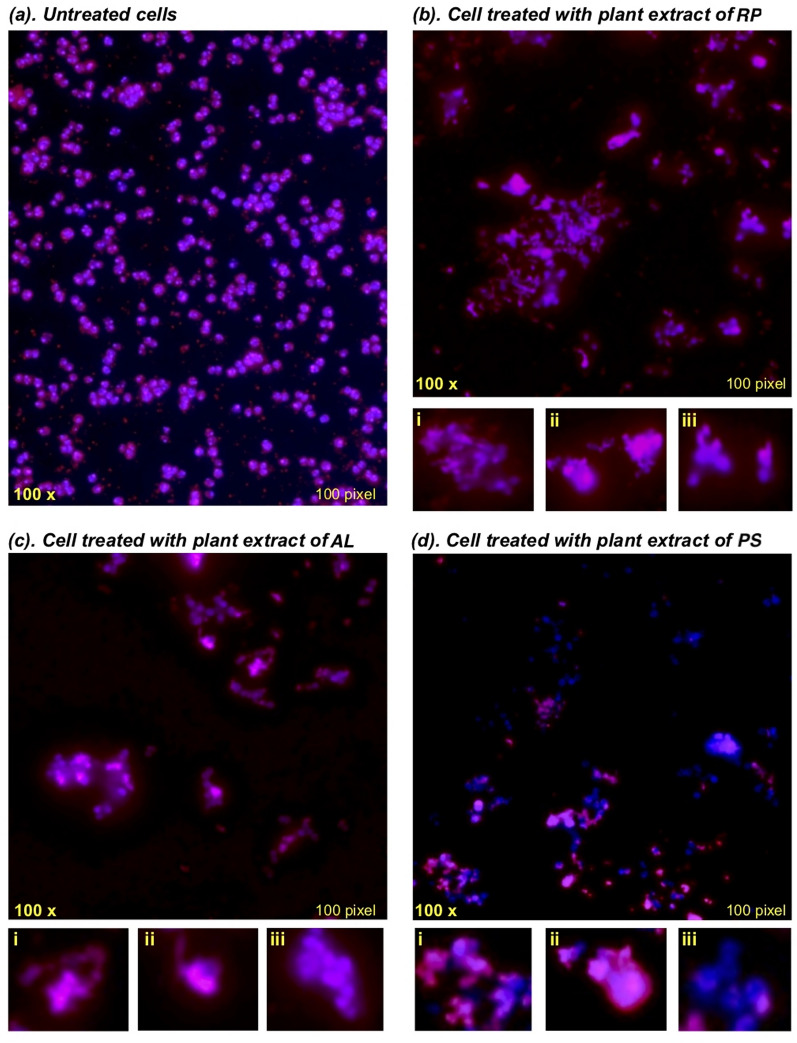
Fluorescence images of MRSA cells after treatment with plant extracts of *R. palmatum (RP)*, *A. lappa (AL)* and *P. suffructicosa (PS)*. FM 4–64 (red) for cell membrane and Hoechst 33342 (blue) for the DNA, in the absence (**a**) or presence of plant extracts at MIC (7.8 μg/ml) for 20 h (**b**–**d**). The images were captured under the magnification of 100 × to illustrate various types of damage to the bacterial cells. (i) The zoomed typical cells of irregular shape. (ii) The zoomed representative cells of losing membrane integrity including the changes of disassociated membrane, bulging and invaginations in the membrane. (iii) The zoomed distinctive cells with changes in DNA

MRSA grown in the absence of plant extract exhibited the characteristic spherical and regular shape surrounding by a smooth membrane stained in red with the blue-stained nucleoid DNA evenly distributed in the cytoplasm (Fig. 4a). In contrast, cells exposed to plant extracts displayed marked changes in MRSA including bulging or invagination of membrane (Fig. 4-ii), disassociated or loss of membrane, or irregular blue DNA staining (Fig. 4-iii). Notably, treatment of *R. palmatum* resulted in the greatest cell membrane damage in MRSA compared to *A. lappa* and *P. suffructicosa*.

**Fig. 5:**
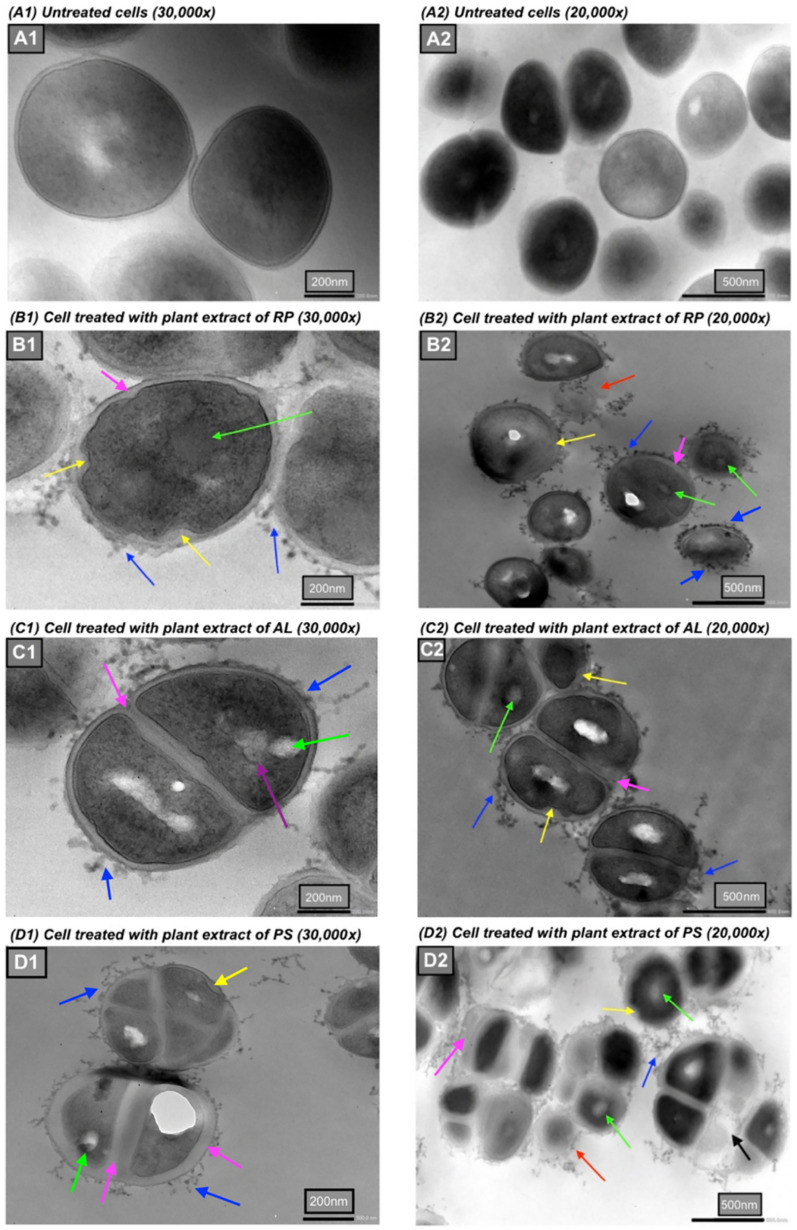
TEM images of MRSA exposed to plant extracts of *R. palmatum (RP)* (B1–2)*, A. lappa (AL)* (C1–2) and *P. suffructicosa (PS)* (D1–2) at their MIC for 20 h. Control: MRSA cells without treatment (A1–2). The images were captured under the magnification of 30,000 × (scale bar = 200 nm) (A1–D1), and 20,000 × (scale bar = 500 nm) (A2–D2). The coloured arrows mark various types of damage in treated bacterial cells, including: thickened septal and peripheral portions of the cell wall (pink arrows), formation of blebs or invagination of cell membrane (yellow arrows), bubbles protruded from cell surface (blue arrows), central condensation (green arrows) and fragmentation (purple arrows) of bacterial DNA, lysed cells (red arrows), and bleb-like gaps between the cell wall and the cytoplasmic membrane (black arrow)

**Fig. 6:**
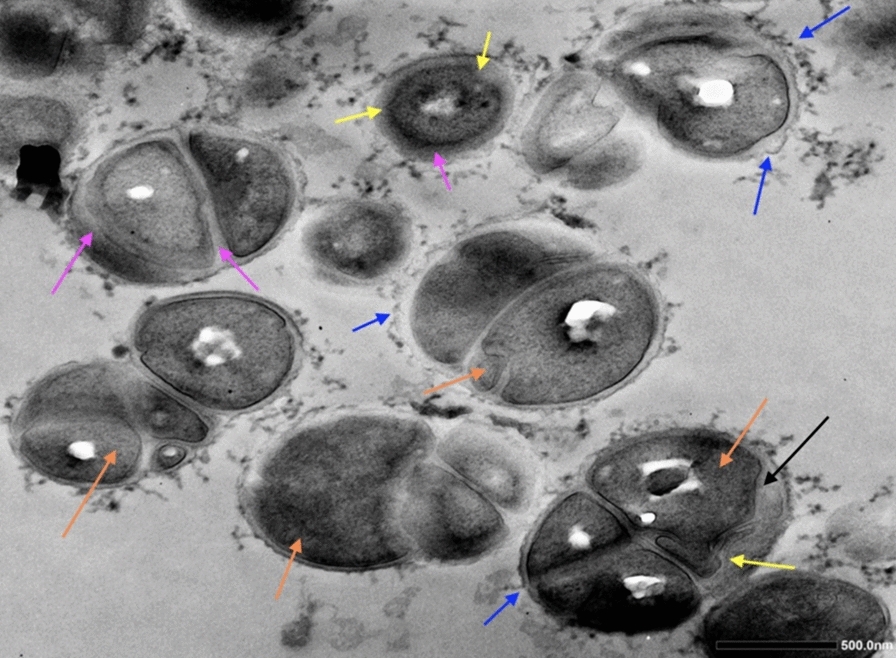
TME micrographs of MRSA exposed to plant extracts of *P. suffructicosa (PS)* at its MIC (7.8 μg/ml) for 20 h. The images were captured under the magnification of 15,000 × (scale bar = 500 nm). MRSA displayed a pseudo-multicellular form as a result of treatment (orange arrow), accompanied with thickened septal and cell wall (pink arrows), bubbles observed outside the cell wall (blue arrows), invagination of the cell membrane (yellow arrows), and the bleb-like gaps between the cell wall and membrane (black arrow)

The TEM micrographs (Fig. 5) unveil several distinct markers denoting the impairment of bacterial cells treated with the plant extracts at their MICs, including significantly thickened cell wall and septal (pink arrows); protruded blebs/blisters outside the cell wall (blue arrows); bugles and invaginations of membrane (yellow arrows); central condensation of nucleoid DNA (green arrows); and the bursting and/or lysing of cells (red arrows). The control cells, on the other hand, retained a unified cell wall and dense cytoplasm structure with non-distinguishable DNA (Fig. 5 A1–A2). The TEM images also reveal a crumpled cytoplasmic membrane after treatment of *R. palmatum* (Fig. 5B) and *A. lappa*, with the latter also accompanied with the central condensed electron-lucent nucleoid DNA appearance (Fig. 5C). Additionally, exposure to *P. suffructicosa* led to the formation of pseudo-multicellular Staphylococci with conspicuous thickened and disrupted cell walls and enlarged cell phenotype compared to the untreated cells (Fig. 5-D2). Fig 6 illustrates a characteristic increase in the prevalence of pseudo-multicellular morphology (orange arrows) in MRSA following treatment with *P. suffructicosa*, displaying various degrees of thickened cell wall (pink arrows) and disruption in the cell membrane (yellow arrows). Table 6 provides a summary of the main morphological changes observed in both FM and TEM images.

**Table 6:** Morphostructural changes in MRSA induced by medicinal plant extracts through FM and TEM analysis

Morphological changes	Cell shape	Cell wall and septal	Cytoplasmic membrane	Cell DNA	Pseudo-multicellular Staphylococci
FM					
Control	Uniform and cocci-shaped	n/a	Intact	Evenly distributed in cytoplasm	n/a
* R.* *palmatum*	Irregular or lysis with debris	n/a	Bulges and invagination	Reduced staining	n/a
* A.* *lappa*	Swollen with debris	n/a	Bulge	Reduced staining	n/a
* P.* *suffructicosa*	Irregular and large	n/a	Bulged and loss of intracellular contents	Condensed and loss of membrane	n/a
TEM					
Control	Uniformly cocci-shaped	Intact	Cytoplasm was homogenously electron dense	Evenly dispersed and non-distinguishable in the cytoplasm	No
* R.* *palmatum*	Swollen, contracted and burst	Thickened and broken, formation of blebs/blisters	Distorted, bugles, invaginations, rough and wrinkled	Condensed	No
* A.* *lappa*	Swollen and enlarged	Thickened and broken, blebs/blisters on wall surface	Distorted and bleb-like structure between cell wall and membrane, and invaginations	Condensed and fragmented	No
* P.* *suffructicosa*	Enlarged and aberrant with multiple cell clusters	Extreme thickness of cell wall and septal, extremely thicken cell wall and septum	Invaginations, bleb-like structure between cell wall and membrane blurred and diffused membrane	Condensed and fragmented	Increased population of pseudo-multicellular phenotype

## Discussion

Owing to Staphylococcus’s intrinsic resistance and the widespread use of antibiotics over the past decades, MRSA has evolved to resist virtually all beta-lactams [13]. As vancomycin is reserved as a last resort treatment for MRSA infections, it is imperative to investigate new and potent antimicrobial agents. The utilisation of traditional herbal medicine for the treatment of infectious diseases has gained global prominence as a leading alternative medicine [14]. Traditional plant species have been extensively examined for their antimicrobial properties using a variety of extraction methods, although many studies have focused on individual compounds derived from medicinal plants. A notable level of antibacterial potency is indicated when the MIC of natural products falls below 1 mg/mL [15].

Antimicrobial agents kill or inhibit bacteria primarily by disrupting their membranes or by interacting with intracellular components, thereby chemically with synthesis or functioning. Consequently, this affects bacterial cell-wall biosynthesis, membrane permeability, and DNA replication and repair [16]. Studies have shown that *R. palmatum* possesses antibacterial properties against a wide range of Gram-positive and Gram-negative bacteria, including *Escherichia coli*, *S. aureus* and MRSA [17]. Aloe-emodin, a prominent phytoconstituents found in *R. palmatum* extracts, inhibited the growth of *S. aureus* by affecting the permeability of the cell membrane, which was evidenced by the reduction of intracellular contents in treated bacterial cells and the disruption of membrane integrity, impacting on components such as phosphatidylethanolamine and phosphatidylglycerol [18]. Isopanduratin A, one of the main flavonoids present in *A. lappa*, induced damage to the cell wall of Gram-positive bacteria, such as *Streptococcus mutans* [19]. Qian and colleagues demonstrated that the phenolic compound paeonol, an active constituent of *P. suffructicosa*, effectively combated *Klebsiella pneumoniae* and *Enterobacter cloacae,* displaying a MIC of 64 μg/mL. It disrupted the integrity of bacterial cell membranes, resulting in cytoplasm leakage, as confirmed by the field emission scanning electron microscopy [20]. The present study is the first to report morphological and ultrastructural changes of MRSA induced by the crude aqueous extracts of *R. palmatum, A. lappa* and *P. suffructicosa* which were produced in a way resembling the traditional preparation of medicinal plants.

In this study, diverse crude plant extracts demonstrated low remarkably low MIC values ranging from 7.8 µg/mL to 31.2 µg/mL, comparable to the antibiotic control of vancomycin (MIC 2 µg/mL). Specifically, *R. palmatum, A. lappa and P. suffructicosa* extracts exhibited the strongest antibacterial activity with the lowest MIC (7.8 μg/mL) and MBC (31.2 μg/mL). The time-kill curves illustrated the dosage and time-dependent efficacy of these three plants. Notably, *P. suffructicosa* at 3 × MIC (i.e. 23.4 µg/mL) exerted a rapid and persistent bactericidal impact, eradicating over 99.9% (> 3-log) of MRSA within 6 h (Fig. 2c). A prior study showed that aqueous extracts of *R. palmatum* inhibited the growth of both Gram-positive bacteria (e.g. *S. aureus*) and Gram-negative bacteria (e.g. *E. coli*) [21]. These results underscore the antibacterial potential of these plants, emphasizing their promising role as antimicrobials.

In addition to evaluating the antibacterial activity of individual medicinal extracts, we explored the interaction between plant substances and therapeutic antibiotics. *R. palmatum*, *A. pilosa*, and *R. arnebiae* exhibited a synergistic effect when combined with vancomycin. Significantly, at their respective MICs, these plant extracts demonstrated the capability to reduce the MIC of vancomycin against MRSA by a factor of four. A comparable effect has been noted with catechin, a flavonoids compound present in plants such as *R. palmatum*, *Cyrtomium fortunei* J. Smith and *Agrimonia pilosa* Ledeb [22, 23]. Catechin has demonstrated diverse effects on the tested examined *S. aureus* strains. A decrease in MIC values of up to two-fold was observed for vancomycin in the presence of catechin [24]. Taylor et al. reported its mechanism of action, involving the disruption of the cell wall and depolarisation of the bacterial cytoplasmic membrane [25]. To our knowledge, this is the first report of synergism between vancomycin and medicinal plants *R. palmatum*, *A. pilosa* and *R. arnebiae*, respectively. Antibiotics have been found to be more effective when combined with plant-derived compounds capable of inhibiting efflux pump proteins and/or inactivating enzymes involved in multidrug resistance [26]. Synergistic mechanism of plant-derived substances and antibiotics may arise from the disruption of the bacterial cell membrane and cell wall facilitated by the phytochemicals, consequently increasing the influx of antibiotics into bacterial cells [27]. The results in this study reinforce the notion that this phenomenon could lead to effective treatments for infections caused by antibiotic-resistant bacteria. In contrast to single-component antibiotics, medicinal plants encompass a diverse array of bioactive phytochemicals, making it more challenging for microbes to adapt and develop resistance [28]. Further research on medicinal plants is required to comprehensively understand the synergistic mechanism, laying the foundation for developing pharmaceutical drugs derived from medicinal plants to control bacterial infections effectively.

Adverse effects stemming from cytotoxicity represent a significant concern in therapeutic drug especially when plant-based therapies are under consideration. In the present study, crude aqueous extracts from eight promising plants demonstrated significant safety profiles for both HpeG2 and Caco-2 cells at concentrations as high as 16 × MIC, with exceptions of *C. fortune* showing weak cytotoxicity towards HepG2 at 16 × MIC (72%), and *A. Pilosa* on Caco-2 with cell viability of 67% at 4 × MIC (Table 5). According to ISO 10993-5 standards, these eight tested plants would not be classified as potential cytotoxic substances, as their viability at MIC is greater than 80%, with the exception of *A. pilosa* (75%) [29]. Traditional medicine integrates plant species with inherent toxic properties, which are counteracted or detoxified by other herbal components. While toxicity may lead to different levels of adverse or undesirable effects, these may not necessarily be lethal [30]. Nonetheless, the challenge arises from insufficient comprehension of the in vivo biokinetic behavior of compounds, preventing the direct utilization of in vitro toxicity data to assess and extrapolate compound toxicity across entire organisms [31]. Consequently, future research endeavors will need to include in vivo toxicity investigations specifically focused on the examined medicinal plant extracts.

Cell membranes serve as a selective barrier, controlling the passage of molecules and ions from the extracellular environment to maintain cellular homeostasis [32]. Upon exposure to the plant extracts at their MICs, both FM and TEM images disclosed the formation of extensive and distinct bulges and invagination. Among them, *R. palmatum* induced the most substantial damage to the membrane (yellow arrows in B1 and B2 of Fig. 5), manifested by the presence of rough, wrinkled membrane, as well as retracted and ruptured cells and cell lysis (red arrow).

W﻿ith its intricate multicomponent structure, the bacterial cell wall plays a crucial role in maintaining the physical architecture necessary to preserve the shape, size, and overall integrity of bacterial cells. Staphylococcal peptidoglycan (PG), the major component of the cell wall, features pentaglycine cross-bridges of nascent peptides that provide mechanical strength and flexibility for bacterial growth in the presence of countering osmotic pressure [33]. Impeding peptidoglycan biosynthesis or destabilizing its integrity can arrest cell growth, as bacterial biological pathways are intricately interlinked, and disruption in one system inevitably affects numerous other functional mechanisms with the cell [34]. In this study, the exposure of MRSA to crude plant extracts resulted in a significantly thickening of the bacterial cell wall. Notable, *P. suffructicosa* induced the most pronounced thickening in both cell wall and the septal, which are characteristics of RNA and/or protein synthesis inhibitors [35]. β-lactam antibiotics, such as imipenem and oxacillin, can induce cell wall thickening by inhibiting peptidoglycan cross-linking, resulting in the accumulation of loose and non-structural cell wall material. This association has also been observed between cell wall thickening and decreased peptidoglycan hydrolase activity [36]. Importantly, recent studies have confirmed that *S. aureus* cells cannot survive without a pentaglycine cross-bridge [37]. Moreover, an intriguing morphological transformation observed in MRSA in our study was the emergence of blebs/vesicles outside the cell wall upon treatment with *R. palmatum* and *A. lappa* (Fig. 6, blue arrows). Formation of blebs, seen as protrusions found in the outer membrane of gram-negative species, has been reported following treatment with membrane-active agents such as peptides. The abundance of bleb-like structures witnessed in MRSA in this study might be attributed to the bacterial defence mechanism against membrane damage and cell lysis caused by plant extracts [38, 39].

Besides impairing cell membranes and walls, the antibacterial impact of plant extract was observed internally within bacterial cells, evidenced by nucleoid condensation in the cytoplasm. It is worth noting that the capability of plant substances to induce alteration in nucleoid DNA has not been documented in previous literature. As illustrated in Fig. 4A, the DNA in MRSA is typically evenly dispersed and indistinguishable in the cytoplasm. However, following treatment with plant extract, the nucleoid DNA exhibited condensation (indicated by green arrows) or fragmentation (purple arrow) in Fig. 5. Nucleoid fragmentation has been reported to coincide with impaired chromosome segregation in *S. aureus* after treatment with nalidixic acid—a quinolone-based antimicrobial compound that damages DNA by targeting the DNA gyrase enzyme [40]. Our discovery of alterations in the nucleoid DNA in addition to cell walls and membranes serves as compelling proof of the multifaceted effects of plant-derived phytochemicals, akin to the impacts of antibiotics and antibacterial peptides.

Remarkably, the application of *P. suffructicosa* treatment led to the formation of pseudo-multicellular MRSA, featuring an unusually thickened cell wall and septum, along with conspicuous bleb-like gaps that separated the cell envelope from the cytoplasm. This finding emphasizes the profound structural changes induced by *P. suffructicosa* on MRSA. Pseudo-multicellular staphylococci has been found in *S. aureus* treated with low concentrations of chloramphenicol and some beta-lactam antibiotics, probably a result of inhibitions of autolytic wall enzymes that typically facilitate cell separation [41]. Similarly, exposure to subinhibitory concentration of vancomycin was observed to swiftly and completely suppress the autolytic system of *S. aureus*, resulting in a transit pseudo-multicellular form [42]. In this study, we discovered that *P. suffructicosa* extract was effective in induced the pseudo-multicellular phenotype after 20 h of treatment at MIC against MRSA. Recognizing the potential of this plant for monotherapy or in combination with vancomycin in managing vancomycin-resistant and other serios infections is a significant factor. The finding may offer insights into the observed rapid and sustained bactericidal activity of *P. suffructicosa*, as reflected in the time-kill curves.

Moreover, it has been shown that inactivation of methicillin resistance genes (*fem*) encoded for FemA and FemB proteins that are essential for pentaglycine bridge synthesis, can give rise to varying forms of pseudo-multicellular morphology. Specifically, the *femA* mutant MRSA exhibited irregularly shaped multiple cells, whereas the *femB* mutant displayed a more regularly arranged pseudo-multicellular phenotype, similar to our observations, suggesting a potential role of *P. suffructicosa* on the *femB* gene. More recently, Monteiro et al*.* confirmed that the emergence of pseudo-multicellular forms in *femAB* depleted *S. aureus* is associated with substantial membrane rupture, ultimately leading to bacterial lysis [37]. Together, it is conceivable to speculate that the anti-MRSA activity of *P. suffructicosa* may be attributed to its distinctive mechanistic action, which involves modulating gene expression, inhibiting DNA replication, and disrupting cell separation.

Our study’s findings unequivocally demonstrated the multifaceted impact of crude medicinal plant extracts prepared in a way that resembles their traditional usage. The coexistence of diverse active phytocomponents in plant-based therapeutics may lead to multifactorial effects, especially when these bioactive compounds act on various bacterial targets or synergize to improve the bioavailability of each constituent [43]. Surmounting AMR requires novel antibacterial agents that can prevail the resistant mechanisms of bacteria and/or resensitize the efficacy of existing antibiotics. The capability of plants to multitarget is crucial in treating multidrug-resistant (MDR) bacterial infections. More importantly, the superiority of phytotherapy is further emphasized by its lower tendence to elicit resistance, a fact that is attested by its longstanding effectiveness in the annals of traditional medicine. Moreover, there is a growing realisation that the conventional approach may not be the most appropriate for advancing phytobiotic developments, which involves bioassay-guided separation, purification, and isolation of individual specific bioactive compound to elucidate their precise mechanisms against resistant bacteria.

In conclusion, the findings presented in this paper offer robust evidence supporting the potential of medicinal plants as effective phytobiotics against resistant bacteria, either independently or in combination with existing antibiotics. This represents a pivotal advancement in phytotherapeutic research, providing a foundation for further exploration and development of botanical alternatives to address the escalating challenge of antimicrobial resistance in the healthcare landscape. Future research endeavours should focus on evaluating the antimicrobial potential of the promising medicinal plants against various MRSA strains, including clinical isolates, to enhance our understanding of their effectiveness. Additionally, conducting further in vivo cytotoxicity and antibacterial assessments towards different strains and clinical isolates will be crucial for developing therapeutic agents that can combat antibacterial resistance and enhance treatment outcomes.

## Data Availability

Data and materials presented in this study are available on request from the corresponding author. Data sharing is not applicable to this article as no datasets were generated or analyzed during the current study.
